# Are Team Sports Effective in Improving Mental Health? A Systematic Review on the Primary Outcomes of Anxiety and Mood

**DOI:** 10.2174/0117450179471779260627171341

**Published:** 2026-07-02

**Authors:** Augusto Cezar Rodrigues Rocha, Alberto Souza Sá Filho, Marcelo Couto Jorge Rodrigues, José Carlos Pontes Corrêa, Matias Noll, Filipe Manuel Clemente, Mário Hebling Campos, Vicente Aprigliano, Sérgio Machado, Carlos Alexandre Vieira, Gustavo De Conti Teixeira Costa

**Affiliations:** 1 Faculdade de Educação Física e Dança, Universidade Federal de Goiás, Goiânia, Brazil; 2 Graduate Program in Human Movement and Rehabilitation, Evangelical University of Goiás – UniEVANGÉLICA, Anápolis, Goiás, Brazil; 3 Graduate Program in Pharmaceutical Sciences, Pharmacology and Therapeutics (PPGCFFT), Evangelical University of Goiás – UniEVANGÉLICA, Anápolis, Goiás, Brazil; 4 Instituto Federal de Educação, Ciência e Tecnologia Goiano, Ceres, Brazil; 5 Gdansk University of Physical Education and Sport, 80-336 Gdańsk, Poland; 6 Sport Physical Activity and Health Research & Innovation Center, Rio Maior, Portugal; 7 Escuela de Ingeniería de Construcción y Transporte, Pontificia Universidad Católica de Valparaíso, Avda Brazil 2147, Valparaíso, Chile; 8 Laboratory of Physical Activity Neuroscience, Neurodiversity Institute, Queimados- RJ , Brazil; 9 LABPR – Panic and Respiration Laboratory (LABPR) - Institute of Psychiatry (IPUB), Federal University of Rio de Janeiro (UFRJ), Rio de Janeiro, Brazil

**Keywords:** Adult, Exercise therapy, Mental health, Quality of life, Social support, Psychosocial outcomes

## Abstract

**Introduction:**

Team Sports (TSG) have been discussed as an accessible and socially engaging way to support mental health. Although this idea is intuitively appealing and supported by several observational findings, the actual consistency of their effects on anxiety and mood in adults who do not have diagnosed mental disorders remains uncertain.

The study aims to map how TSG has been investigated in this context and to systematically synthesize current evidence on their effects on anxiety and mood (PROSPERO: CRD420251121611).

**Methods:**

This systematic review followed PRISMA 2020 recommendations. Five databases were searched: PubMed, Scopus, Web of Science, EMBASE, and Cochrane Library. Eligible studies included longitudinal, face-to-face TSG interventions involving adults or older adults and reporting validated psychometric outcomes. Randomized trials were assessed using the Cochrane RoB 2 tool and the PEDro scale, whereas non-randomized studies were evaluated using ROBINS-I.

**Results:**

Six studies involving 744 participants met the inclusion criteria. The included interventions involved soccer/football, floorball, volleyball/soccer programs, small-sided soccer games, and recreational TSG. Two studies reported reductions in anxiety symptoms, while one randomized trial showed reduced mood disturbance when small-sided games were combined with verbal encouragement. One trial found no significant changes in generalized anxiety, depressive symptoms, perceived stress, or well-being. Another randomized trial showed that recreational TSG improved mental-health-related quality of life compared with control.

**Discussion:**

Evidence suggests that TSG may promote improvements in symptoms of anxiety and depression. However, in non-clinical adult populations, the value of TSG may lie less in producing large reductions in psychiatric symptoms and more in improving psychosocial functioning, perceived well-being, and social connection.

**Conclusion:**

Current evidence suggests that TSG may improve selected mental-health and psychosocial outcomes in adults, including anxiety symptoms, mood disturbance, emotional well-being, and mental-health-related quality of life. However, these effects were inconsistent among studies and were strongly influenced by intervention context.

## 1 INTRODUCTION

Chronic diseases, including cardiovascular disease, diabetes, cancer, chronic respiratory disease, and mental disorders, are the leading causes of mortality worldwide [1-3]. Especially, the risk of mental health problems is closely associated with lifestyle and social determinants, while chronic physical conditions may further increase psychiatric vulnerability [3-6]. The high prevalence of mental disorders has placed unprecedented pressure on health systems, exposing the limitations of traditional models of care [4, 7]. As a result, strategies aimed at behavioral change have gained increasing attention, particularly those involving regular physical exercise [8-15].

Regular exercise has been consistently associated with reductions in symptoms of mental disorders, especially depression and anxiety [8, 9, 11-14, 16-23]. These benefits are partly explained by neurobiological mechanisms, including the release of neurotransmitters such as endorphins, the attenuation of the Hypothalamic-Pituitary-Adrenal Axis (HPA) activation [24], and the expression of serotonin and Brain-Derived Neurotrophic Factor (BDNF) [25, 26]. Accordingly, physical exercise is recognized as a non-pharmacological therapeutic strategy with relatively few side effects and additional health benefits [27-32].

Sport is one of the principal forms of engagement in physical exercise and has been incorporated into public policies as a strategy for health promotion and disease prevention [17, 18, 33-37]. It has also been increasingly recognized as a viable approach to mental health promotion, given its capacity to reach large and diverse populations [17, 18, 33]. Team Sports (TSG), in particular, tend to produce more favorable mental health outcomes than individual sports, particularly for depression and anxiety, due to increased social interaction, with a sense of belonging and interpersonal communication as underlying mechanisms of the mental health benefits of TSG [33, 38]. TSG also fosters greater intrinsic motivation related to enjoyment and social interaction [39], and improves anxiety-related outcomes [17, 18, 33, 36] while reducing depression [19, 36].

Despite increasing investigation on this topic, studies have focused on elite athletes (6% of the population), which is problematic since 94% of the population does not fall into this category. Additionally, a wide range of existing studies are conducted with populations with different mental disorders, especially mood and anxiety disorders [9, 16, 21, 22, 28, 29, 31]. Therefore, the results of these studies cannot be extrapolated to a segment of the population that does not have a diagnosis, but is affected by chronic stressors and anxiolytics, which can potentially increase symptoms.

To our knowledge, no previous review has specifically synthesized longitudinal and interventional evidence on face-to-face team-sport interventions in adults not recruited on the basis of a formal diagnosed mental disorder. Therefore, this systematic review aimed to synthesize longitudinal evidence on the effects of face-to-face TSG on anxiety, mood, and broader mental-health-related outcomes in adults without a diagnosis of mental disorders. Secondarily, we will analyze aspects of quality of life, perceptions of health, emotional well-being, and sleep quality.

## 2 METHODS

This study was conducted as a systematic review, and the protocol was registered in the PROSPERO database under the number (CRD420251121611) [40]. In accordance with PRISMA 2020 recommendations, we conducted a comprehensive database search, applied explicit eligibility criteria, and performed independent dual-reviewer screening for study selection, data extraction, and quality assessment. Randomized controlled trials were assessed using the Physiotherapy Evidence Database (PEDro scale) [41] and the Cochrane Risk of Bias 2 (RoB 2.0 tool) [42], while non-randomized intervention studies were evaluated using the assessment for non-randomized intervention studies (ROBINS-I framework) [43].

### 2.1 Study Question and Eligibility Criteria

The criteria for conducting the systematic review followed the PICOS guidelines. They were: a) Population (P): Adults and older adults (≥ 18 years), physically able to participate in sports, without medical conditions that prevent participation; b) Intervention (I): Regular practice of TSG, either recreational or semi-professional; c) Comparator (C): Control groups not engaged in structured physical exercise or practicing non-team modalities, such as individual aerobic exercise; resistance training; or other interventions; Outcomes (O): Primary: reduction of anxiety symptoms and improvement of mood indicators, assessed with validated psychometric instruments (*e.g.*, POMS); Secondary: improvements in subjective well-being, quality of life, and social support indicators; Study design (S): only longitudinal Randomized Controlled Trials (RCT), or longitudinal quasi-experimental studies were considered, published in English or Portuguese, with no date restriction.

Studies were excluded if they met any of the following conditions: a) populations with clinical diagnoses of depression, anxiety disorders, bipolar disorder, psychotic disorders, or other psychiatric conditions under active medical treatment (to avoid clinical confounding not attributable to the sports intervention); b) interventions conducted within rehabilitation, therapy, or clinical treatment programs, including hospital-, psychiatric-, or psychotherapy-based sport programs; c) studies analyzing virtual, simulated, exergame-based, or electronic (e-sport) team sports, as these do not meet the criteria of face-to-face cooperative physical participation; d) cross-sectional, purely observational, or qualitative studies without a longitudinal or intervention component; e) studies that did not include validated psychometric instruments for assessing anxiety, mood, or other mental-health-related outcomes; f) studies involving youth (< 18 years), adolescents, or elite/high-performance athletes, due to motivational, physiological, and psychosocial profiles distinct from the general adult population; g) reviews, opinion papers, theoretical essays, conference abstracts, dissertations, or non-peer-reviewed publications.

### 2.2 Information Sources and Search

A comprehensive literature search was conducted in PubMed (MEDLINE), Embase (Elsevier), Web of Science (Core Collection), Scopus, and the Cochrane Library, without restrictions on date or language. The search strategy was broad to maximize sensitivity and identify all potentially relevant studies on team sports and mental-health-related outcomes in adults. Because the field overlaps with clinical psychiatry, rehabilitation, youth sport, and observational sport psychology, many records retrieved in the initial search were expected to fall outside the final eligibility criteria.

The comprehensive electronic search was finalized on October 28, 2025, and all records available up to that date were included for screening and eligibility assessment. Search terms were combined with Boolean operators (AND, OR, NOT) and adjusted to fit the specific syntax requirements of each database. The search strategies are presented in Table **1**, and the following keyword combination was used: team sport; team game; sport team; group sport; anxiety; depression; mood; mental health; adult; older adult; elderly.

### 2.3 Outcome Variables

The primary outcomes extracted were anxiety and mood, considered core indicators of adult mental health and the most frequently assessed emotional dimensions in exercise and sport science research. Anxiety was defined as self-reported symptoms of tension, worry, and physiological arousal, assessed using validated instruments such as the Generalized Anxiety Disorder Scale (GAD-7), the Hospital Anxiety and Depression Scale (HADS), and context-specific social anxiety measures. Mood was defined as a multidimensional affective state encompassing transient positive and negative emotions and was primarily assessed using the Profile of Mood States (POMS) or related instruments (*e.g.*, Brunel Mood Scale [BRUMS]), which quantify Total Mood Disturbance (TMD) and subdomains including vigor, tension, fatigue, and depression.

Because the included literature assessed mental health using heterogeneous constructs, secondary outcomes were extracted when they reflected broader mental-health-related or psychosocial domains. These included depressive symptoms, post-traumatic stress symptoms, perceived stress, psychological well-being, emotional well-being, mental-health-related quality of life, self-efficacy, sleep-related outcomes, relationship satisfaction, parental stress, and family functioning. Instruments considered eligible included, but were not limited to, the Patient Health Questionnaire-9 (PHQ-9), Impact of Event Scale-Revised (IES-R), Perceived Stress Scale (PSS), WHO-5 Well-being Index, Short-Form Health Survey mental-health components, RAND-36, Older People’s Quality of Life questionnaire, General Self-Efficacy Scale, Satisfaction With Life Scale, Parental Stress Scale, Relationship Assessment Scale, and Family Inventory measures.

### 2.4 Selection of Sources of Evidence

Manuscript selection was performed using Rayyan (Qatar Computing Research Institute) reference management software, through which duplicate records were removed. Two reviewers (AR and MR) independently conducted the initial screening by reading titles and abstracts according to the inclusion and exclusion criteria. Discrepancies were resolved through discussion, and when consensus could not be reached, a third reviewer (GC) made the final decision. Full-text screening followed the same procedure. During full-text assessment, special attention was given to four decision domains (in this order): population eligibility, intervention format (TSG), study design (longitudinal), and outcome validity. Articles failing in any of these domains were excluded. The selection spreadsheet is presented in the **supplementary material**.

### 2.5 Data Charting Process and Data Items

A data extraction spreadsheet was developed to collect information from the studies included in this review. Diverse information was extracted from the manuscripts and encompassed the following items: a) Bibliographic information (title, author, year of publication, country, and journal); b) manuscript information (study objective, sample, study type, team sport used, and mechanisms for controlling levels of anxiety and mood disorders); c) characteristics of instruments used to assess anxiety and mood disorders (instrument name and frequency of use).

This extraction and comparison were conducted independently by two authors (AR and MR) using the completed spreadsheet. Discrepancies were resolved through discussion; when critical information was unclear or missing, corresponding authors of the original studies were contacted for clarification.

### 2.6 Synthesis of Results

The extracted information was organized and presented through summary tables and figures, accompanied by a narrative synthesis that helped contextualize the scope and main characteristics of the evidence. To give structure to the material, data were grouped into thematic categories such as study selection, publication features, intervention characteristics, and mental-health–related outcomes (including session frequency, program duration, measurement tools, and the overall direction of effects).

Before synthesizing the findings, we standardized the reported data to enhance comparability among studies. Missing data were not imputed; when essential information was unclear, we contacted the corresponding authors for clarification.

The main results were presented in structured tables (*e.g.*, Tables **1**–**3**) that summarized the study design, intervention characteristics, and observed outcomes. Risk-of-bias evaluations were illustrated using RoB 2 and ROBINS-I traffic-light plots. Additional figures depicted the study selection pathway (PRISMA flow), publication trends over time, and the geographic distribution of included research, facilitating comparisons across diverse study designs and contexts.

A narrative synthesis was employed to integrate the findings, as the included studies varied substantially in design (randomized *vs.* non-randomized), intervention structure, competitive *vs.* recreational context, sample characteristics, and outcome measurement instruments. These factors limited the methodological comparability required for a valid meta-analysis.

### 2.7 Quality of Evidence and Risk of Bias Assessment

The PEDro scale and risk of bias assessment were conducted independently by two reviewers, with disagreements resolved by consensus.

The methodological risk of bias of included studies was independently assessed by two reviewers using the Cochrane Collaboration’s Risk of Bias tools. To do this, we use the following tools: a) the RoB 2.0 tool and the ROBINS-I tool. Each domain was evaluated individually, and studies were categorized as having low risk of bias, some concerns, or high risk of bias.

The methodological quality of the randomized controlled trials included in this review was assessed using the PEDro scale. The PEDro scale is a validated instrument designed to evaluate the internal validity and statistical reporting quality of clinical trials involving physical exercise and rehabilitation. The instrument consists of 11 items, although the first item (eligibility criteria) relates to external validity and is therefore not included in the final score, resulting in a scoring range of 0 to 10 points.

Each criterion was scored as present (1 point) or absent (0 points), and the sum of all applicable items generated the final PEDro score for each trial. Based on established interpretation guidelines, total scores were classified as excellent (9–10 points), good (6–8 points), moderate (4–5 points), or poor methodological quality (≤ 3 points).

## 3 RESULTS

### 3.1 Study Selection

**Fig. (1):**
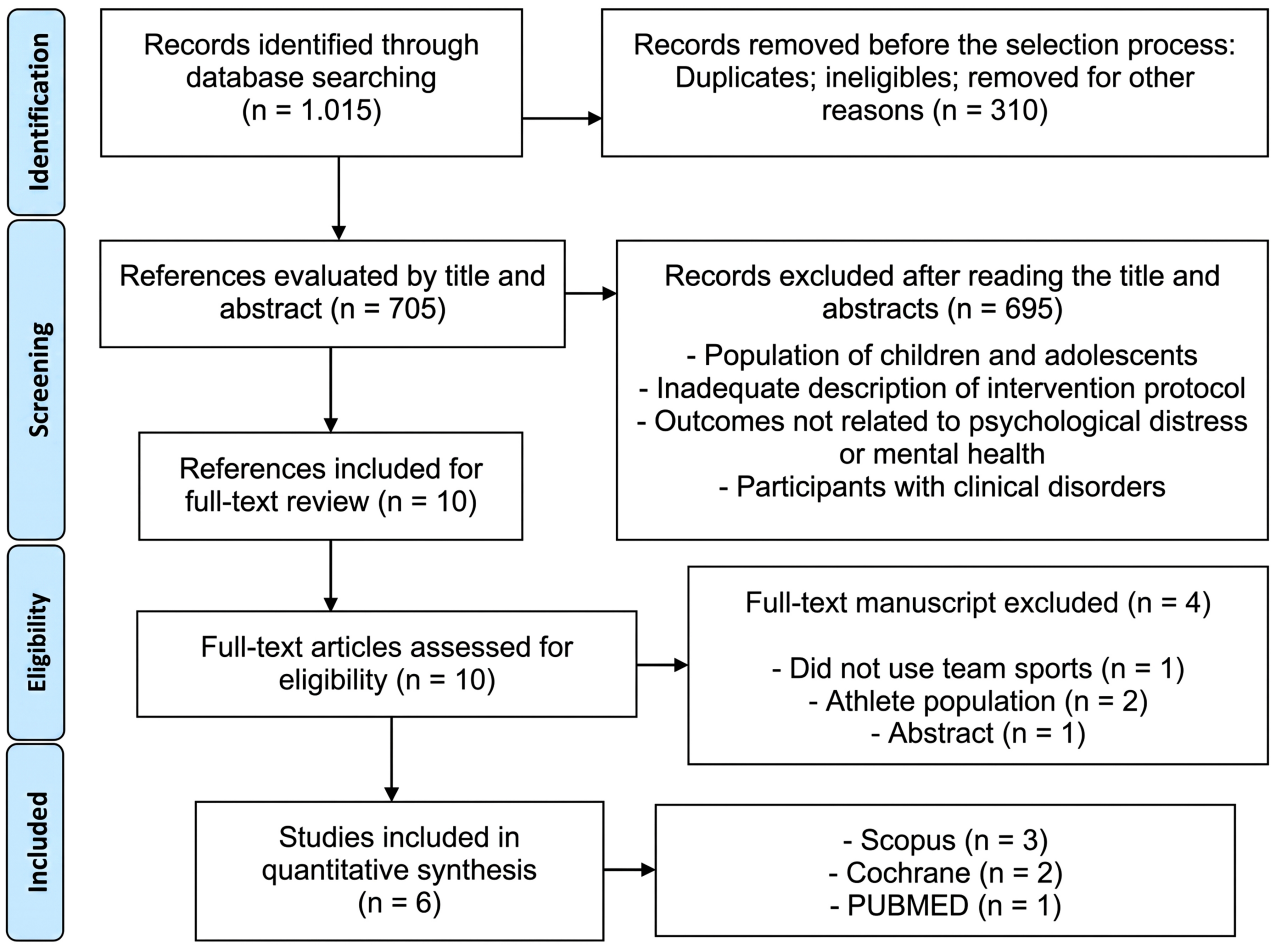
Flow diagram of study search, screening, and selection.

The disposition of search results is presented in Fig. (**1**) as a PRISMA flow diagram. Searching the five databases using the selected keywords yielded 1,015 manuscripts. Of these, 310 were excluded as duplicates, resulting in 705 titles for screening. After title screening, 695 manuscripts were excluded for failing to meet the inclusion criteria, and 9 manuscripts proceeded to full-text assessment. Of the 9 full-text articles assessed for eligibility, 3 were excluded mainly because they involved clinical psychiatric populations, non-face-to-face or non-team-sport interventions, or the athlete population. This screening process resulted in the final inclusion of only six manuscripts in the review (Fig. **1**). The selection and exclusion results are presented in Tables **S1** and **S2** (**Supplementary Material**).

Due to the substantial heterogeneity among the included studies, mainly study design, sport modality, intervention frequency, total duration, comparator type, participant profile, and competitive context, meta-analysis was not considered appropriate. Instead, the findings were synthesized narratively. In this synthesis, studies were compared according to key intervention characteristics in order to examine whether differences in format, exposure, and context could plausibly explain variability in anxiety- and mood-related outcomes.

### 3.2 Publication Data and Geographic Location of Publications

The included studies were published between 2017 and 2025. Only 2 studies were published between 2017 and 2021 with participants without specific mental disorders, whereas in the last years, *i.e.*, from 2021 onward, there was a greater concentration of selected manuscripts.

The included studies were conducted in different countries, and international collaborations were observed, involving partnerships among countries such as Denmark [39], China/USA [36], Canada [44], Switzerland/Greece [35], Norway/Denmark [45], Tunisia/Turkey/Portugal [37].

### 3.3 Population, type of Study, and Team-sport Modalities

A total of 744 participants were assigned to intervention groups with TSG, an alternative intervention, or a control. The smallest sample included (n= 13), and the largest, n = 153. The experimental group had a total of 318 participants (42,7%), as shown in Table **2**.

### 3.4 Instruments used for Data Collection

Mental-health-related outcomes were assessed using a heterogeneous set of validated psychometric instruments, reflecting the broad conceptual scope of anxiety, mood, perceived stress, well-being, quality of life, and psychosocial functioning. Mood-related outcomes were mainly assessed with the Profile of Mood States (POMS), which was used by Romdhani *et al.* [37] to examine total mood disturbance and specific mood dimensions in semi-professional soccer players. In addition, the Satisfaction Scale for Athletes was applied in the same study to assess sport-related satisfaction alongside mood responses.

Anxiety and depressive symptoms were assessed with different instruments across studies. Pedersen *et al.* [39] used the Danish version of the Hospital Anxiety and Depression Scale (HADS), and Johnston *et al.* [36] assessed depressive symptoms using the Center for Epidemiologic Studies Depression Scale Revised (CESD-R) and anxiety symptoms using the Generalized Anxiety Disorder Scale (GAD-7). Similarly, Filippou *et al.* [35] used the GAD-7 to assess anxiety symptoms and the Patient Health Questionnaire-9 (PHQ-9) to assess depressive symptoms among asylum seekers living in a camp.

Post-traumatic stress symptoms were assessed only in Filippou *et al.* [35] using the Impact of Event Scale-Revised (IES-R), which evaluates intrusion, avoidance, and hyperarousal symptoms. The same study also assessed perceived stress using the Perceived Stress Scale-10 (PSS-10) and psychological well-being using the WHO-5 Well-being Index. Perceived stress was also assessed in Johnston *et al.* [36], although the specific stress-related instrument was reported as the Perceived Stress Scale.

Quality of life, health perception, self-efficacy, sleep, and broader psychosocial functioning were assessed as secondary or complementary outcomes. Pedersen *et al.* [39] evaluated quality of life using the Danish version of the Older People’s Quality of Life questionnaire and the SF-12, whereas self-perceived health was reported but not sufficiently described in methodological detail. Barene and Krustrup [45] assessed self-reported health status and emotional well-being using the RAND-36 Health Survey, and general self-efficacy using the General Self-Efficacy Scale (GSE-6). This study also included sleep-related outcomes, such as poor and restless sleep, difficulty falling asleep, waking too early, waking several times during the night, and total sleep problems.

Rhodes *et al.* [44] expanded the psychosocial scope of the review by assessing mental health as the primary outcome using the six-item mental-health subscale of the Short-Form 12 Health Survey (SF-12). Secondary outcomes included overall life satisfaction, assessed with the Satisfaction With Life Scale; parental stress, assessed with the Parental Stress Scale; relationship satisfaction, assessed with the Relationship Assessment Scale; and family functioning, assessed with subscales of the Family Inventory Version II, including family health/competence, low conflict, cohesion, and emotional expressiveness.

### 3.5 Quality of Evidence and Risk of Bias

The risk-of-bias assessment was conducted independently by two reviewers, with disagreements resolved by consensus. Randomized trials were assessed using the RoB 2 tool, whereas non-randomized controlled intervention studies were assessed using the ROBINS-I tool. This distinction was based on the allocation procedure rather than the mere presence of a comparison group. Therefore, studies with a control group but without a clearly reported random allocation procedure were treated as non-randomized intervention studies and evaluated using ROBINS-I (Table **3A** and **3B**).

Among the randomized trials assessed with RoB 2, three studies were judged to have some concerns overall: Barene and Krustrup [45], Rhodes *et al.* [44], and Romdhani *et al.* [37]. These judgments were mainly driven by methodological limitations that are common in exercise and behavioral intervention trials, including lack of participant blinding, reliance on self-reported psychometric outcomes, and uncertainty or incomplete reporting regarding allocation concealment and outcome-assessor blinding.

Filippou *et al.* [35] were judged as having a high overall risk of bias. Although the study used a pragmatic randomized controlled design, approximately one-third of randomized participants did not complete the post-intervention assessment. In addition, attrition was not entirely random, as participants who completed the trial differed from those who did not complete it in baseline mental-health outcomes. This raised major concerns in the missing outcome data domain. Although this adherence-based analysis was clinically informative and consistent with the intervention rationale, it depends on post-randomization participation frequency and therefore weakens causal interpretation.

Rhodes *et al.* [44] were judged as having some concerns rather than a high risk of bias. The study used a three-arm randomized design, included active and non-active comparison conditions, and analyzed repeated observations using generalized linear mixed models. However, participants could not be blinded to allocation, the outcomes were self-reported, and retention at the 3-month endpoint was incomplete. These issues justify caution in interpretation, even though the study design and statistical handling of repeated measures were stronger than those of several other included studies.

The two non-randomized studies assessed with the ROBINS-I tool, Pedersen *et al.* [39] and Johnston *et al.* [36], were judged to have a high overall risk of bias. The main concerns were related to baseline confounding and participant selection, since group allocation was not clearly randomized and pre-existing differences between groups could not be ruled out as explanations for observed changes. Although both studies used defined interventions and validated psychometric instruments, the lack of random allocation, limited control for confounding, and reliance on self-reported outcomes reduced confidence in causal interpretation.

### 3.6 Synthesis of Results

The six studies included in this review differed considerably in study design, participant characteristics, intervention format, sport modality, comparator type, intervention dose, and outcome measures. Four studies were randomized controlled trials, whereas two used non-randomized or quasi-experimental longitudinal designs. The TSG interventions ranged from structured small-sided soccer games with semi-professional players to recreational team sport participation among parents, floorball in older adults, workplace soccer among hospital employees, mixed team-sport programs among college students, and a pragmatic exercise-and-sport program for asylum seekers living in a camp.

Intervention duration ranged from 6 weeks to 40 weeks. Frequency also varied substantially, from once weekly in some recreational or community-based formats to daily availability in the pragmatic camp-based intervention. The comparator conditions included inactive control groups, waiting-list control, individual aerobic exercise, resistance training, Zumba, and non-active personal-time activities.

Regarding anxiety-related outcomes, the findings were heterogeneous. Pedersen *et al.* [39] reported reductions in anxiety symptoms among older adults participating in floorball, while Johnston *et al.* [36] observed reductions in anxiety among college students engaged in team sports, although changes were not clearly superior to aerobic exercise. Filippou *et al.* [35] found no significant improvement in generalized anxiety symptoms assessed with the GAD-7, despite improvements in PTSD symptoms among participants who attended the exercise and sport program at least twice per week. Rhodes *et al.* [44] did not use a specific anxiety scale but reported improvements in the SF-12 mental-health component, which includes domains related to vitality, social functioning, emotional well-being, depressive symptoms, and anxiety symptoms. These findings suggest that team-sport interventions may benefit anxiety-related dimensions, but the effect is not consistent among populations or instruments.

Mood and depressive outcomes also showed mixed results. Romdhani *et al.* [37] reported a reduction in total mood disturbance only in the small-sided games condition with coaches’ verbal encouragement, suggesting that the social and motivational climate of the intervention may influence mood responses. Pedersen *et al.* [39] observed reductions in depressive symptoms among older adults, and Johnston *et al.* [36] reported reductions in depressive symptoms among college students. In contrast, Filippou *et al.* [35] did not observe significant changes in depressive symptoms assessed with the PHQ-9. Barene and Krustrup [45] did not primarily assess depression or mood disturbance, but reported changes in emotional well-being and general self-efficacy.

For post-traumatic stress symptoms, only Filippou *et al.* [35] assessed this outcome. In the overall intervention-*versus*-control comparison, the time-by-condition interaction was not significant. However, participants who attended the exercise and sport activities at least twice per week showed a significant reduction in PTSD symptoms, whereas no change was observed among those attending less frequently or in the waiting-list control group.

Secondary psychosocial outcomes were reported in several studies and provided additional context for interpreting the mental-health effects of TSG participation. Rhodes *et al.* [44] found that recreational TSG participation improved mental health and relationship satisfaction compared with individual physical activity and a non-active personal-time control condition. TSG also reduced parental stress and improved family emotional expressiveness compared with the control condition. Barene and Krustrup [45] reported improvements in emotional well-being and general self-efficacy among hospital employees participating in football training, although several sleep-related outcomes remained unchanged. Pedersen *et al.* [39] reported improvements in quality-of-life domains and self-perceived health among older adults.

The direction and magnitude of effects appeared to depend strongly on context. Programs delivered in socially supportive and recreational environments tended to show more favorable responses in anxiety, mood, well-being, or quality of life. Conversely, effects were weaker or more inconsistent in studies with lower adherence, higher contextual stress, or more competitive/performance-oriented characteristics.

Finally, Barene and Krustrup [45] and Romdhani *et al.* [37] were both rated as moderate quality on the PEDro scale, each scoring 5/10. Filippou *et al.* [35] scored 3/10, indicating poor methodological quality, mainly due to unclear allocation concealment, baseline imbalance, lack of blinding, incomplete follow-up, and absence of a clearly reported intention-to-treat analysis. Rhodes *et al.* [44] scored 6/10, indicating moderate methodological quality, reflecting its randomized three-arm design, allocation procedures, use of repeated-measures statistical models, between-group comparisons, and reporting of estimates, although the score was limited by lack of participant/provider blinding and incomplete follow-up.

The two non-randomized studies, Pedersen *et al.* [39] and Johnston *et al.* [36], were not scored with PEDro because their allocation procedures were not clearly randomized. These studies were assessed using ROBINS-I and were judged as having a high overall risk of bias, mainly because of potential baseline confounding and participant selection bias. Although both studies included comparison groups and used validated psychometric instruments, the absence of random allocation limited causal interpretation.

Table **4** presents the characteristics of the studies included in the sample, as well as a summary of the outcomes observed.

## 4 DISCUSSION

This systematic review synthesized longitudinal evidence on the effects of face-to-face team-sport interventions on anxiety, mood, and broader mental-health-related outcomes in adults not recruited on the basis of a formal diagnosis of mental disorder. The findings suggest that TSG may contribute to selected improvements in anxiety symptoms, total mood disturbance, and, secondarily, to emotional well-being, mental-health-related quality of life, self-efficacy, relationship satisfaction, parental stress, and quality-of-life domains. However, these effects were inconsistent across studies and should be interpreted cautiously due to the small number of eligible trials, marked heterogeneity in intervention design, differences in participant characteristics, and moderate to high risk of bias.

The main contribution of this review is that it narrows the interpretation of sport and mental-health research to a specific intervention format: longitudinal, face-to-face TSG participation. Previous reviews have generally examined broader categories of sport participation, physical activity, or exercise, often combining observational and interventional designs, clinical and non-clinical populations, youth and adult samples, and individual and team-based modalities [23, 33, 34]. In contrast, the present review focused on whether TSG interventions themselves appear to produce favorable mental-health responses in adults without formal psychiatric recruitment. This distinction is important because the potential benefit of TSG is not only physiological, as with other forms of exercise, but also social and contextual. TSG may provide social interaction, shared goals, group identity, interpersonal communication, enjoyment, and perceived belonging, which are mechanisms frequently proposed to explain why sport participation may be associated with better mental health [33, 34, 46].

The findings partially support this hypothesis [11, 14, 15, 46, 47]. Anxiety-related outcomes improved in some studies, particularly in older adults participating in floorball and in college students engaged in TSG activities [36, 39]. These results are consistent with broader evidence showing that physical activity and exercise can reduce anxiety symptoms in adult populations [9, 12-14, 16, 48, 49]. However, the effects were not uniform. Filippou *et al.* [35] did not find significant improvements in generalized anxiety symptoms despite implementing a pragmatic exercise-and-sport intervention in a highly vulnerable population of asylum seekers [35]. This suggests that the anxiolytic effects of TSG may not be automatic and may depend on population context, baseline psychosocial burden, adherence, safety, and the stability of the surrounding environment. In populations exposed to substantial chronic stressors, such as forced displacement or uncertainty regarding asylum status [35], the psychosocial load may be strong enough to attenuate the psychological benefits usually expected from exercise or sport participation.

Mood-related outcomes were also inconsistent. Romdhani *et al.* [37] reported reduced total mood disturbance only when small-sided soccer games were combined with coaches’ verbal encouragement [37]. This finding is important because it suggests that the emotional effect of TSG may depend not only on the sport modality but also on the motivational climate in which the activity occurs. Verbal encouragement may increase perceived support, enjoyment, engagement, and competence, thereby improving affective responses during and after participation. This interpretation aligns with theoretical models proposing that autonomy, competence, relatedness, and social support are central mechanisms linking sport participation to mental-health benefits [23, 33, 34, 46]. Therefore, TSG should not be treated as a uniform exposure. The same sport may produce different psychological responses depending on whether it is delivered as a supportive, recreational, inclusive, and socially engaging activity or as a competitive, evaluative, or performance-oriented experience [50, 51].

Evidence from exercise trials often shows larger psychological benefits in individuals with clinically elevated symptoms or diagnosed mood and anxiety disorders [31, 49], likely because higher baseline symptom burden creates greater room for improvement [8, 28, 29, 46]. In contrast, adults without formal diagnoses may present lower symptom levels, making changes harder to detect and more dependent on contextual moderators [11, 19]. In this review, the clearest benefits often appeared in outcomes that were broader than anxiety or mood alone, such as emotional well-being, self-efficacy, relationship satisfaction, parental stress, family emotional expressiveness, and quality of life [39, 44, 45]. This suggests that, in non-clinical adult populations, the value of TSG may lie less in producing large reductions in psychiatric symptoms and more in improving psychosocial functioning, perceived well-being, and social connection.

The study by Rhodes *et al.* [44] is especially relevant in this respect. Although it did not use a disorder-specific scale for anxiety or depression, recreational TSG participation improved mental-health-related quality of life and relationship satisfaction compared with individual physical activity and a non-active personal-time control condition [44]. It also reduced parental stress and improved family emotional expressiveness compared with the control group. These findings strengthen the idea that the social dimension of TSG may produce benefits that are not fully captured by traditional anxiety or depression scales. For parents of young children, participation in sport may provide structured time away from caregiving demands, opportunities for adult social interaction, enjoyment, and a sense of personal identity beyond the parental role.

The heterogeneity observed among studies also reflects a broader reporting problem in TSG intervention research. TSGs are complex interventions, and their mental-health effects may depend on the prescription setting. However, these parameters were rarely objectively monitored. As a result, it is difficult to determine whether inconsistent findings are attributable to true differences in intervention effectiveness or contextual characteristics of the participants. This is particularly important for anxiety and mood outcomes, which may be influenced by both physiological arousal and the social-emotional environment of the intervention. Future studies should therefore follow reporting frameworks such as CERT and TIDieR to improve reproducibility, comparison across trials, and translation into practice [52, 53].

It is worth highlighting that among the sport modalities identified in the included studies, soccer/football was the most frequently represented, either as the main intervention or as one component of broader TSG programs. This predominance may reflect the global reach, consolidated organizational structure, and extensive competitive infrastructure of soccer/football, supported by international governing bodies such as FIFA and UEFA [54]. These factors may facilitate participant recruitment, collaborations, and research visibility, thereby contributing to the greater scientific representation of soccer/football compared with other TSG [54].

Finally, regular participation in sports may modulate neuroendocrine responses, including the HPA axis, reduce cortisol reactivity, and influence neurotransmitters and neuropeptides related to mood regulation, such as dopamine, serotonin, and endorphins [24, 27]. Regular physical activity may also contribute to lower chronic low-grade inflammation and modulate inflammatory mediators associated with depressive and anxiety symptoms [55, 56]. However, these mechanisms were not directly tested in the included studies. Therefore, they should be interpreted as biologically plausible explanations rather than conclusions supported by the reviewed trials. From a psychosocial perspective, TSG may promote mental health through social support, group cohesion [24, 25, 27, 30, 32], shared identity, perceived competence, enjoyment, and structured interpersonal engagement [23, 33, 34]. These mechanisms may be especially relevant in non-clinical populations, in which changes in well-being and social functioning may be more detectable than changes in clinical symptom scores.

## 5 LIMITATIONS OF EVIDENCE

The small number of eligible studies and the wide variation in their methods make it difficult to draw firm conclusions about the effects of TSG on anxiety and mood. Differences in participant characteristics, the structure of the interventions, and the length and frequency of the programs create a fragmented evidence base. Without greater standardization, comparing results across studies becomes challenging, which ultimately weakens the strength of the inferences that can be made.

An important limitation of the available evidence concerns the risk of bias and confounding within the primary studies. Two of the included studies used non-randomized or quasi-experimental designs, which increases vulnerability to selection bias and baseline confounding. In these studies, pre-existing group differences cannot be ruled out as partial explanations for the observed changes in anxiety, depression, or related psychosocial outcomes. Even in the randomized trials, important methodological limitations remained, particularly unclear allocation concealment, absence of blinding, and incomplete protection against performance and detection bias. These issues are especially relevant in exercise-based behavioral interventions, in which participants are usually aware of the intervention they receive. The populations studied also restrict the scope of the findings. Much of the evidence comes from university students, semi-professional athletes, or older adults, with little representation of community-based, culturally diverse, or clinically vulnerable groups. This narrow sampling makes it difficult to understand how team sports might function as a mental-health strategy in real-world public health settings.

Finally, none of the studies included long-term follow-up assessments, which prevents us from knowing whether the observed benefits persist over time or fade once the intervention ends. Future research would benefit from extended follow-up periods to clarify whether the effects of TSG on mental health are short-lived, sustained, or cumulative.

## 6 LIMITATIONS OF THE REVIEW PROCESSES

Although we carried out a broad and thorough search across major databases, we did not systematically review grey literature, preprints, or other non-indexed sources. This gap raises the possibility that some unpublished or ongoing studies may not have been captured. Study selection and data extraction were conducted independently by two reviewers, but certain steps, such as interpreting narrative results or classifying mental-health constructs, still involve an unavoidable degree of subjective judgment.

## 7 IMPLICATIONS FOR PRACTICE AND FUTURE RESEARCH

Studies should prioritize better intervention standardization. In particular, studies should provide more standardized descriptions of the intervention, including sport modality, session frequency, session duration, total program length, progression, and degree of supervision. Exercise intensity and competitive load should also be reported more explicitly, as these variables were largely absent or insufficiently described in the included studies and may directly influence anxiety- and mood-related responses.

In addition, future research should prioritize adequately powered randomized controlled designs, improved baseline comparability between groups, and longer follow-up periods to determine whether observed benefits persist after the intervention ends. Recruitment should also extend beyond narrow samples such as university students, semi-professional athletes, or specific occupational groups, with greater emphasis on community-based and more diverse adult populations. These improvements are necessary to strengthen causal inference, increase external validity, and clarify under which conditions team sports may meaningfully support mental health.

Recent psychometric research also reinforces the need for greater methodological rigor in the selection and adaptation of mental-health instruments across contexts. For example, the development and validation of the Healthcare Worker Stress Scale-Vietnamese followed a structured process that included forward-backward translation, expert review, content validity testing, exploratory and confirmatory factor analysis, and reliability assessment, illustrating good practice in cross-cultural adaptation and construct validation. Although developed for occupational stress in healthcare workers, this study highlights an important methodological direction for future team-sport trials: psychological outcomes should not be treated as interchangeable among settings without adequate cultural and psychometric support. This is particularly relevant when mood, anxiety, perceived stress, and well-being are assessed in diverse adult populations using instruments originally developed in other languages or contexts [57].

## CONCLUSION

Current evidence suggests that TSG may improve selected mental-health and psychosocial outcomes in adults, including anxiety symptoms, mood disturbance, emotional well-being, mental-health-related quality of life, relationship satisfaction, parental stress, self-efficacy, and quality-of-life domains. However, these effects were inconsistent among studies and were strongly influenced by intervention context, adherence, population characteristics, comparator type, and outcome measures. Therefore, TSG should be considered a promising, but not yet established, strategy for mental-health promotion in adults without diagnosed mental disorders.

## Figures and Tables

**Table 1:** Search strategy among different databases.

PUBMED	(“team sport*” OR “team game*” OR “sport team” OR “group sport*”) AND (“anxiety” OR “depression” OR “mood” OR “mental health”) AND (“adult*” OR “older adult*” OR “elderly”) NOT (“adolescent” OR “teenager” OR “youth” OR “child”)
Web of Science	team sport (Topic) and sport (Topic) and anxiety (Topic) and depression (Topic) and mood (Topic) not adolescents (Topic)
Scopus	TITLE-ABS-KEY ((“team sport*” OR “team game*” OR “sport team” OR “group sport*”) AND (“anxiety” OR “depression” OR “mood” OR “mental health”) AND (“adult*” OR “older adult*” OR “elderly”) AND NOT (“adolescent” OR “teenager” OR “youth” OR “child”))
EMBASE	('team sport*':ti,ab,kw OR 'team game*':ti,ab,kw OR 'sport team':ti,ab,kw OR 'group sport*':ti,ab,kw) AND ('anxiety':ti,ab,kw OR 'depression':ti,ab,kw OR 'mood':ti,ab,kw OR 'mental health':ti,ab,kw) AND ('adult*':ti,ab,kw OR 'older adult*':ti,ab,kw OR 'elderly':ti,ab,kw) NOT ('adolescent':ti,ab,kw OR 'teenager':ti,ab,kw OR 'youth':ti,ab,kw OR 'child':ti,ab,kw)
Cochrane Library	#1 (“team sport*” OR “team game*” OR “sport team” OR “group sport*”) #2 (“anxiety” OR “depression” OR “mood” OR “mental health”) #3 (“adult*” OR “older adult*” OR “elderly”) #4 (“adolescent” OR “teenager” OR “youth” OR “child”) #5 #1 AND #2 AND #3 NOT #4 “team sport” in Title Abstract Keyword OR “team game” OR “group sport” OR “team game” in Title Abstract Keyword AND anxiety OR depression OR mood OR “mental health” in Title Abstract Keyword AND adult OR “older adult” OR elderly in Title Abstract Keyword NOT adolescent OR teenager OR youth OR child in Title Abstract Keyword

**Table 2:** A comprehensive description of the population investigated.

**Study**	**Design of Study**	**Participants and (Sex)**
Pedersen *et al.* [39]	Longitudinal non-randomized CT	TSG:13 (9 women); RT: 19 (10 women); CG = 12 (6 women);
Johnston *et al.* [36]	Longitudinal quasi-experimental	TSG: 138 (67 women); AE: 153 (all women);
Barene and Krustrup [45]	Longitudinal RCT	TSG: 35 (women); ZG: 34 (women); CG: 34 (women);
Filippou *et al.* [35]	Longitudinal RCT	TSG: 49 (29 women); CG: waiting-list - 49 (28 women);
Rhodes *et al.* [44]	Longitudinal RCT, three-arm parallel design	TSG: 58 (38 women); ISG: 60 (40 women); CG: 66; (44 women);
Romdhani *et al.* [37]	Longitudinal RCT	TSG with verbal encouragement: 14 (only men); TSG without verbal encouragement: 15 (only men); CG: 14 (only men);

**Abbreviations:** TSG: Team Sport Group; RT: Resistance Training; AE: Aerobic Training; CG: Control Group; ZG: Zumba Group; ISG: Individual Physical Activity; CT: Controlled Intervention.

**Table 3A:** Risk of bias assessment ROB2.

**Study**	**D1**	**D2**	**D3**	**D4**	**D5**	**Overall** **Judgment**
Barene and Krustrup [45]	Some concerns	Some concerns	Some concerns	Some concerns	Some concerns	Some concerns
Filippou *et al.* [35]	Some concerns	Some concerns	High risk	Some concerns	Some concerns	High risk
Rhodes *et al.* [44]	Some concerns	Some concerns	Some concerns	Some concerns	Low risk	Some concerns
Romdhani *et al.* [37]	Some concerns	Some concerns	Some concerns	Some concerns	Some concerns	Some concerns

**Table 3B:** Risk of bias assessment ROBINS-I tool.

**Study**	**D1**	**D2**	**D3**	**D4**	**D5**	**D6**	**D7**	**Overall Judgment**
Pedersen *et al.* [39]	High risk	High risk	Low risk	Some concerns	Some concerns	Some concerns	Some concerns	High risk
Johnston *et al.* [36]	High risk	High risk	Low risk	Some concerns	Some concerns	Some concerns	Some concerns	High risk

**Table 4:** Participant characteristics, description of the interventions, and direction of outcomes.

**Author [ref.]** **Classification**	**Population** **Age (years)**	**Intervention Description**	**Frequency and Duration**	**Anxiety and Mood Outcomes** **(Direction of Change)**
Romdhani *et al.* [37]; PEDro 5	Semi-professional soccer; TSG1: 23.3 ± 2.1 TSG2: 24.7 ± 2.2 CG: 24.5 ± 1.7	Small-sided games with VE *vs.* without VE *vs.* CG.	10 sessions in 6 weeks	POMS: ↓ TMD (only TSG1 reduced); SSA: ↑ (TSG1 and TSG2, but not for CG);
Filippou *et al.* [35]; PEDro 3	Asylum seekers living in a camp; TSG: 29.87 ± 10.12 years.	Football, volleyball, basketball, martial arts, *vs.* aerobics and dance.	10 weeks (5 days/week); 1-h sessions/day, separated by sex.	IES-R: ↓ PTSD symptoms only among participants attending ≥2 sessions/week; PHQ-9: ↔ depressive symptoms; GAD-7: ↔ anxiety symptoms; PSS-10: ↔ perceived stress; WHO-5: ↔ well-being.
Barene and Krustrup [45]; PEDro 5	Hospital employees; TSG: 44.8 ± 8.9 ZG: 46.3 ± 9.5 CG: 47.2 ± 9.4	TSG (Soccer) *vs.* ZG vs CG.	3× (12 weeks) and 2× (28 weeks); Total = 40 weeks	↔ Self-Reported Health Status (only ZG ↑ in 12 and 40 weeks); ↑ Emotional well-being (NS among TSG/ZG); ↑ General Self-Efficacy (only TSG); Sleep: ↔ poor and restless sleep (only ZG reduced); ↔ problems falling asleep; ↔ Woke up too early without falling asleep again; ↔ Woke up several times and unable to fall asleep again; ↔ total score of sleep problems.
Rhodes *et al.* [44]; PEDro 6	Parents with children under 13 years living at home. TSG: 37.70 ± 7.90; IPA: 38.16 ± 6.27; CG: 37.37 ± 7.26.	TSG: Basketball, soccer, volleyball, kickboxing classes, running groups *vs.* CG: Going for coffee, going to the movies, taking an arts and crafts workshop	3 months; at least 1 session/week; ≥ 1 h/session.	SF-12 mental health: ↑ TSG *vs.* individual PA and date-night control at 3 months; RAS: ↑ relationship satisfaction; Parental Stress Scale: ↓ parental stress *vs.* control; Family Inventory: ↑ emotional expressiveness *vs.* control. Satisfaction with life improved over time across conditions, with no specific condition effect.
Johnston *et al.* [36]; PEDro NA	College students; TSG: 18.9 ± 1.2 AE: 18.3 ± 1.2	TSG (Volleyball/soccer) *vs.* AE	90 minutes weekly 12 weeks	↓Anxiety (NS among groups); ↓Depression; ↑ Stress (NS among groups); ↑ Sleep quality; (NS among groups)
Pedersen *et al.* [39]; PEDro NA	Older adults TSG: 79.0 ± 7.2 RT: 79.2 ± 6.6 CG: 81.3 ± 5.1	Floorball team *vs.* RT	2x/week for 12 weeks	Psychological health: (↓ anxiety; ↓ depression); Self- perceived health: (↑ Psychological well-being; ↔ Psychological limitations; ↔ Psychological well- being; ↔ Social well- being; ↔ Energy; ↔ General health); QoL: (↑ health related; ↑ general; ↔ social; ↔ emotional).

**Abbreviations:** VE = verbal encouragement; TMD = total mood disorder score; SSA = Satisfaction Scale for Athlete; TSG1 = team sport with verbal encouragement; TSG2 = team sport without verbal encouragement; ZG = zumba group; AE = aerobic group; TSG = team sport group; RT = resistance training group; CG = control group; QoL = quality of life; NS = Not significant; PEDro = quality rating scale; PEDro NA = not subject to evaluation; IPA = individual physical activity; RAS = Relationship Assessment Scale.

## Data Availability

All the data and supportive information are provided within the article.

## LIST OF ABBREVIATIONS

AE: Aerobic Training
BRUMS: Brunel Mood Scale
CG: Control Group
CESD-R: Center for Epidemiologic Studies Depression Scale
GAD-7: Generalized Anxiety Disorder Scale
HADS: Hospital Anxiety and Depression Scale
NS: Not Significant
POMS: Profile of Mood States
PSS: Perceived Stress Scale
QoL: Quality of Life
RCT: Randomized Controlled Trials
RT: Resistance Training Group
SSA: Satisfaction Scale for Athlete
TMD: Total Mood Disturbance
TSG: Team Sports
TSG1: Team Sport With Verbal Encouragement
TSG2: Team Sport Without Verbal Encouragement
VE: Verbal Encouragement;
ZG: Zumba Group

## STANDARDS OF REPORTING

PRISMA guidelines and methodology were followed.

## SUPPLEMENTARY MATERIAL

PRISMA checklist is available as supplementary material on the publisher’s website along with the published article. Supplementary material is available on the publisher’s website along with the published article.
