# Supplementary material for: Are Team Sports Effective in Improving Mental Health? A Systematic Review on the Primary Outcomes of Anxiety and Mood
Source: Clin Pract Epidemiol Ment Health. 2026 Jul 2;22:e17450179471779. doi: 10.2174/0117450179471779260627171341 (PMC13458477; doi:10.2174/0117450179471779260627171341)
Supplement: Supplementary file 1 [file CPEMH-22-E17450179471779_SD1.pdf]

Clin. Pract. Epidemiol. Ment. Health
ISSN: 1745-0179
DOI: 10.2174/0117450179471779260627171341, 2026, 22, e17450179471779
1
SUPPLEMENTARY MATERIAL
OPEN ACCESS

# Are Team Sports Effective in Improving Mental Health? A Systematic Review on the Primary Outcomes of Anxiety and Mood

Augusto Cezar Rodrigues Rocha[1,#], Alberto Souza Sá Filho[2,3,*,#], Marcelo Couto Jorge Rodrigues[1], José Carlos Pontes Corrêa[1], Matias Noll[4], Filipe Manuel Clemente[5,6], Mário Hebling Campos[1], Vicente Aprigliano[7], Sérgio Machado[3,8,9], Carlos Alexandre Vieira[1] and Gustavo De Conti Teixeira Costa[1,#]

[1]*Faculdade de Educação Física e Dança, Universidade Federal de Goiás, Goiânia, Brazil*
[2]*Graduate Program in Human Movement and Rehabilitation, Evangelical University of Goiás – UniEVANGÉLICA, Anápolis, Goiás, Brazil*
[3]*Graduate Program in Pharmaceutical Sciences, Pharmacology and Therapeutics (PPGCFFT), Evangelical University of Goiás – UniEVANGÉLICA, Anápolis, Goiás, Brazil*
[4]*Instituto Federal de Educação, Ciência e Tecnologia Goiano, Ceres, Brazil*
[5]*Gdansk University of Physical Education and Sport, 80-336 Gdańsk, Poland*
[6]*Sport Physical Activity and Health Research & Innovation Center, Rio Maior, Portugal*
[7]*Escuela de Ingeniería de Construcción y Transporte, Pontificia Universidad Católica de Valparaíso, Avda Brazil 2147, Valparaíso, Chile*
[8]*Laboratory of Physical Activity Neuroscience, Neurodiversity Institute, Queimados-RJ, Brazil*
[9]*LABPR – Panic and Respiration Laboratory (LABPR) - Institute of Psychiatry (IPUB), Federal University of Rio de Janeiro (UFRJ), Rio de Janeiro, Brazil*

© 2026 The Author(s). Published by Bentham Open.
This is an open access article distributed under the terms of the Creative Commons Attribution 4.0 International Public License (CC-BY 4.0), a copy of which is available at: https://creativecommons.org/licenses/by/4.0/legalcode. This license permits unrestricted use, distribution, and reproduction in any medium, provided the original author and source are credited.

*Address correspondence to this author at the Graduate Program at the Evangelical University of Goiás (UniEVANGÉLICA). Av. Universitária, s/n - Cidade Universitária, Anápolis, Goiás, Brazil;
E-mail: doutor.alberto@outlook.com
[#]*The authors equally contributed to the development of this work.*

Published: July 02, 2026

*Cite as:* Rocha A, Sá Filho, Rodrigues M, Corrêa J, Noll M, Clemente F, Campos M, Aprigliano V, Machado S, Vieira C, Costa G. Are Team Sports Effective in Improving Mental Health? A Systematic Review on the Primary Outcomes of Anxiety and Mood. Clin Pract Epidemiol Ment Health, 2026; 22: e17450179471779.
http://dx.doi.org/10.2174/0117450179471779260627171341

***Send Orders for Reprints to reprints@benthamscience.net***

**Table S1. List of included articles, as well as the reasons.**

| Author | Title | Year | Source | Status | Observations | Decision |
|---|---|---|---|---|---|---|
| Rhodes *et al.*, 2025 [44] | Effect of recreational sport and physical activity participation on well-being during early parenthood: a randomized controlled trial | 2025 | Cochrane and EMBASE | Y | Related | Confirmed |
| Pedersen *et al.*, 2017 [39] | Effect of team sports and resistance training on physical function, quality of life, and motivation in older adults | 2017 | Cochrane and Scopus and PUBMED | Y | Related | Confirmed |
| Filippou *et al.*, 2025 [35] | Effects of an exercise and sport intervention on symptoms of post-traumatic stress disorder and other mental health indices among asylum seekers in a camp. A pragmatic randomized controlled trial | 2025 | Scopus | Y | Related | Confirmed |

| Author | Title | Year | Source | Status | Observations | Decision |
|---|---|---|---|---|---|---|
| Johnston *et al.,* 2021 [36] | Effects of team sports on anxiety, depression, perceived stress, and sleep quality in college students | 2021 | Scopus | Y | Related | Confirmed |
| Barene and Krustrup, 2022 [45] | Football and Zumba Training in Female Hospital Staff: Effects after 12 and 40 Weeks on Self-Reported Health Status, Emotional Wellbeing, General Self-Efficacy and Sleep Problems | 2022 | PUBMED | Y | Related | Confirmed |
| Romdhani *et al.,* 2024 [37] | Small-sided-games with coaches' verbal encouragement have a positive effect on aerobic performance, mood state, satisfaction and subjective effort in male semi-professional soccer players | 2024 | Scopus | Y | Related | Confirmed |

## Table S2. List of excluded articles, as well as the reasons.

| Author | Title | Year | Source | Status | Observations | Decision |
|---|---|---|---|---|---|---|
| Xu and Sun, 2021 | Campus Football Activities On Relieving College Students' Social Anxiety | 2021 | Cochrane | N | Related | Not included. This is a Abstract |
| Fu *et al.* 2025 | Group sports games for social anxiety in university students: a randomized controlled trial revealing gender-specific efficacy | 2025 | Scopus | N | Related | Not included. This is group recreation. |
| Filaire *et al.* 2001 | Preliminary results on mood state, salivary testosterone:cortisol ratio and team performance in a professional soccer team | 2001 | PUBMED | N | Related | Not Included. Athletes population |

## Prisma Checklist.

| Section and Topic | Item # | Checklist item | Location where item is reported |
|---|---|---|---|
| **TITLE** | | | |
| Title | 1 | Identify the report as a systematic review. | reported |
| **ABSTRACT** | | | |
| Abstract | 2 | See the PRISMA 2020 for Abstracts checklist. | Done as required |
| **INTRODUCTION** | | | |
| Rationale | 3 | Describe the rationale for the review in the context of existing knowledge. | Line 77-96 |
| Objectives | 4 | Provide an explicit statement of the objective(s) or question(s) the review addresses. | Line 92-96 |
| **METHODS** | | | |
| Eligibility criteria | 5 | Specify the inclusion and exclusion criteria for the review and how studies were grouped for the syntheses. | Line 138-168 |
| Information sources | 6 | Specify all databases, registers, websites, organisations, reference lists and other sources searched or consulted to identify studies. Specify the date when each source was last searched or consulted. | Line 180-190 |
| Search strategy | 7 | Present the full search strategies for all databases, registers and websites, including any filters and limits used. | Line 191-194 |
| Selection process | 8 | Specify the methods used to decide whether a study met the inclusion criteria of the review, including how many reviewers screened each record and each report retrieved, whether they worked independently, and if applicable, details of automation tools used in the process. | Line 219-226 |
| Data collection process | 9 | Specify the methods used to collect data from reports, including how many reviewers collected data from each report, whether they worked independently, any processes for obtaining or confirming data from study investigators, and if applicable, details of automation tools used in the process. | Line 228-238 |
| Data items | 10a | List and define all outcomes for which data were sought. Specify whether all results that were compatible with each outcome domain in each study were sought (e.g. for all measures, time points, analyses), and if not, the methods used to decide which results to collect. | Line 196-217 |
| | 10b | List and define all other variables for which data were sought (e.g. participant and intervention characteristics, funding sources). Describe any assumptions made about any missing or unclear information. | Line 151-162 |
| Study risk of bias assessment | 11 | Specify the methods used to assess risk of bias in the included studies, including details of the tool(s) used, how many reviewers assessed each study and whether they worked independently, and if applicable, details of automation tools used in the process. | Line 275-282 |
| Effect measures | 12 | Specify for each outcome the effect measure(s) (e.g. risk ratio, mean difference) used in the synthesis or presentation of results. | Line 242-254 |

*(Table) contd.....*

| Section and Topic | Item # | Checklist item | Location where item is reported |
|---|---|---|---|
| Synthesis methods | 13a | Describe the processes used to decide which studies were eligible for each synthesis (e.g. tabulating the study intervention characteristics and comparing against the planned groups for each synthesis (item #5)). | Line 242-254 |
| | 13b | Describe any methods required to prepare the data for presentation or synthesis, such as handling of missing summary statistics, or data conversions. | Line 254-260 |
| | 13c | Describe any methods used to tabulate or visually display results of individual studies and syntheses. | Line 261-267 |
| | 13d | Describe any methods used to synthesize results and provide a rationale for the choice(s). If meta-analysis was performed, describe the model(s), method(s) to identify the presence and extent of statistical heterogeneity, and software package(s) used. | Line 268-272 |
| | 13e | Describe any methods used to explore possible causes of heterogeneity among study results (e.g. subgroup analysis, meta-regression). | Not required |
| | 13f | Describe any sensitivity analyses conducted to assess robustness of the synthesized results. | Not required |
| Reporting bias assessment | 14 | Describe any methods used to assess risk of bias due to missing results in a synthesis (arising from reporting biases). | Not required |
| Certainty assessment | 15 | Describe any methods used to assess certainty (or confidence) in the body of evidence for an outcome. | Line 283-294 |
| **RESULTS** | | | |
| Study selection | 16a | Describe the results of the search and selection process, from the number of records identified in the search to the number of studies included in the review, ideally using a flow diagram. | Line 298-305 |
| | 16b | Cite studies that might appear to meet the inclusion criteria, but which were excluded, and explain why they were excluded. | Line 298-305 |
| Study characteristics | 17 | Cite each included study and present its characteristics. | Line 311-318 |
| Risk of bias in studies | 18 | Present assessments of risk of bias for each included study. | Line 353-367 |
| Results of individual studies | 19 | For all outcomes, present, for each study: (a) summary statistics for each group (where appropriate) and (b) an effect estimate and its precision (e.g. confidence/credible interval), ideally using structured tables or plots. | Line 412-413 |
| Results of syntheses | 20a | For each synthesis, briefly summarise the characteristics and risk of bias among contributing studies. | Line 376-384 |
| | 20b | Present results of all statistical syntheses conducted. If meta-analysis was done, present for each the summary estimate and its precision (e.g. confidence/credible interval) and measures of statistical heterogeneity. If comparing groups, describe the direction of the effect. | Not required |
| | 20c | Present results of all investigations of possible causes of heterogeneity among study results. | Not required |
| | 20d | Present results of all sensitivity analyses conducted to assess the robustness of the synthesized results. | Not required |
| Reporting biases | 21 | Present assessments of risk of bias due to missing results (arising from reporting biases) for each synthesis assessed. | Not required |
| Certainty of evidence | 22 | Present assessments of certainty (or confidence) in the body of evidence for each outcome assessed. | Line 385-390 |
| **DISCUSSION** | | | |
| Discussion | 23a | Provide a general interpretation of the results in the context of other evidence. | Line 415-424 |
| | 23b | Discuss any limitations of the evidence included in the review. | Limitations of Evidence session |
| | 23c | Discuss any limitations of the review processes used. | Limitations of the Review Processes session |
| | 23d | Discuss implications of the results for practice, policy, and future research. | Implications for Practice and Future Research session |
| **OTHER INFORMATION** | | | |
| Registration and protocol | 24a | Provide registration information for the review, including register name and registration number, or state that the review was not registered. | Line 135 |
| | 24b | Indicate where the review protocol can be accessed, or state that a protocol was not prepared. | Line 136 |
| | 24c | Describe and explain any amendments to information provided at registration or in the protocol. | Not required |
| Support | 25 | Describe sources of financial or non-financial support for the review, and the role of the funders or sponsors in the review. | Properly reported |
| Competing interests | 26 | Declare any competing interests of review authors. | Properly reported |
| Availability of data, code and other materials | 27 | Report which of the following are publicly available and where they can be found: template data collection forms; data extracted from included studies; data used for all analyses; analytic code; any other materials used in the review. | Not required |

*From:* Page MJ, McKenzie JE, Bossuyt PM, Boutron I, Hoffmann TC, Mulrow CD, *et al*. The PRISMA 2020 statement: an updated guideline for reporting systematic reviews. BMJ 2021;372:n71. doi: 10.1136/bmj.n71
For more information, visit: http://www.prisma-statement.org/
